# The mitotic spindle protein SPAG5/Astrin connects to the Usher protein network postmitotically

**DOI:** 10.1186/2046-2530-1-2

**Published:** 2012-04-25

**Authors:** Ferry FJ Kersten, Erwin van Wijk, Lisette Hetterschijt, Katharina Bauβ, Theo A Peters, Mariam G Aslanyan, Bert van der Zwaag, Uwe Wolfrum, Jan EE Keunen, Ronald Roepman, Hannie Kremer

**Affiliations:** 1 Department of Human Genetics, Radboud University Nijmegen Medical Centre, 6500 HB Nijmegen, The Netherlands; 2 Department of Otorhinolaryngology, Head and Neck Surgery, Radboud University Nijmegen Medical Centre, 6500 HB Nijmegen, The Netherlands; 3 Department of Ophthalmology, Radboud University Nijmegen Medical Centre, 6500 HB Nijmegen, The Netherlands; 4 Nijmegen Centre for Molecular Life Sciences, Radboud University Nijmegen Medical Centre, 6500 HB Nijmegen, The Netherlands; 5 Donders Institute for brain, cognition and behaviour, Radboud University Nijmegen Medical Centre, 6500 HB Nijmegen, The Netherlands; 6 Department of Cell and Matrix Biology, Institute of Zoology, Johannes Gutenberg University of Mainz, D-55099 Mainz, Germany; 7 Department of Neuroscience and Pharmacology, Rudolf Magnus Institute of Neuroscience, University Medical Centre Utrecht, 3584 CG Utrecht, The Netherlands; 8 These authors contributed equally to the work

## Abstract

**Background:**

Mutations in the gene for Usher syndrome 2A (*USH2A) *are causative for non-syndromic retinitis pigmentosa and Usher syndrome, a condition that is the most common cause of combined deaf-blindness. To gain insight into the molecular pathology underlying USH2A-associated retinal degeneration, we aimed to identify interacting proteins of USH2A isoform B (USH2A^isoB^) in the retina.

**Results:**

We identified the centrosomal and microtubule-associated protein sperm-associated antigen (SPAG)5 in the retina. SPAG5 was also found to interact with another previously described USH2A^isoB ^interaction partner: the centrosomal ninein-like protein NINL^isoB^. Using *In situ *hybridization, we found that *Spag5 *was widely expressed during murine embryonic development, with prominent signals in the eye, cochlea, brain, kidney and liver. *SPAG5 *expression in adult human tissues was detected by quantitative PCR, which identified expression in the retina, brain, intestine, kidney and testis. In the retina, Spag5, Ush2a^isoB ^and Ninl^isoB ^were present at several subcellular structures of photoreceptor cells, and colocalized at the basal bodies.

**Conclusions:**

Based on these results and on the suggested roles for USH proteins in vesicle transport and providing structural support to both the inner ear and the retina, we hypothesize that SPAG5, USH2A^isoB ^and NINL^isoB ^may function together in microtubule-based cytoplasmic trafficking of proteins that are essential for cilium formation, maintenance and/or function.

## Background

Mutations in the gene for Usher syndrome 2A *(USH2A) *are causative for non-syndromic recessive retinitis pigmentosa (RP) [1-4] and for Usher syndrome type II (USH2), a recessive disease characterized by congenital moderate to severe stable hearing loss, and RP that often leads to blindness [5]. Mutations in this gene probably account for 8 to 20% of the autosomal recessive RP cases [3,6], and are suggested to be the commonest cause of RP in the USA [3]. It is estimated that up to 85% of patients with USH2 and about half of all patients with Usher syndrome have mutations in *USH2A *[7]. All proteins encoded by genes associated with USH1 and USH2 are present in hair cells and photoreceptor cells, and are interconnected in a network of interacting proteins [8-12].

To gain insight into the molecular pathology of retinal degeneration resulting from *USH2A *mutations, we aimed to determine the retinal repertoire of USH2A^isoB^-interacting proteins. By using the intracellular domain of USH2A^isoB ^as bait in an interaction trap screen of a retinal cDNA library expressed in yeast (yeast two-hybrid screening), we recently identified the centrosomal protein NINL^isoB^, previously known as Nlp (ninein-like protein). Ninl^isoB ^colocalized with Ush2a^isoB ^at centrioles, basal bodies and in the periciliary regions of photoreceptor cells [13]. We hypothesized that NINL^isoB ^functions in handing over cargo vesicles from the transport system of the inner segment to the intraflagellar transport (IFT) machinery that is involved in transport through the connecting cilium [13,14]. Thereby, NINL^isoB ^may function in the development and maintenance of the connecting cilium and outer segment [13].

In addition to NINL^isoB^, another centrosomal and microtubule-associated protein was identified in the yeast two-hybrid screen, namely sperm-associated antigen (SPAG)5, also called astrin. SPAG5 was originally identified as a microtubule-associated protein with dual localization to both centrosomes and kinetochores, and is required for mitotic spindle formation and chromosome segregation [15,16]. Targeting of SPAG5 to the centrosome during the S and G2 phases of the cell cycle is mediated by ninein, and the SPAG5-ninein interaction is required for the maintenance of centrosome/spindle pole integrity [17]. Interestingly, ninein is a paralog of NINL, which prompted us to investigate the interaction between SPAG5 and NINL^isoB^. In this study, we describe the specific interaction between SPAG5 and both USH2A^isoB ^and NINL^isoB^, and their (partial) colocalization in photoreceptor cells. Our results suggest that these proteins function directly or indirectly in the microtubule-based vesicle transport that is essential for the long-term maintenance and/or function of photoreceptor cells.

## Results

### Interaction of SPAG5 with USH2A^isoB ^and NINL^isoB^

**Figure 1:**
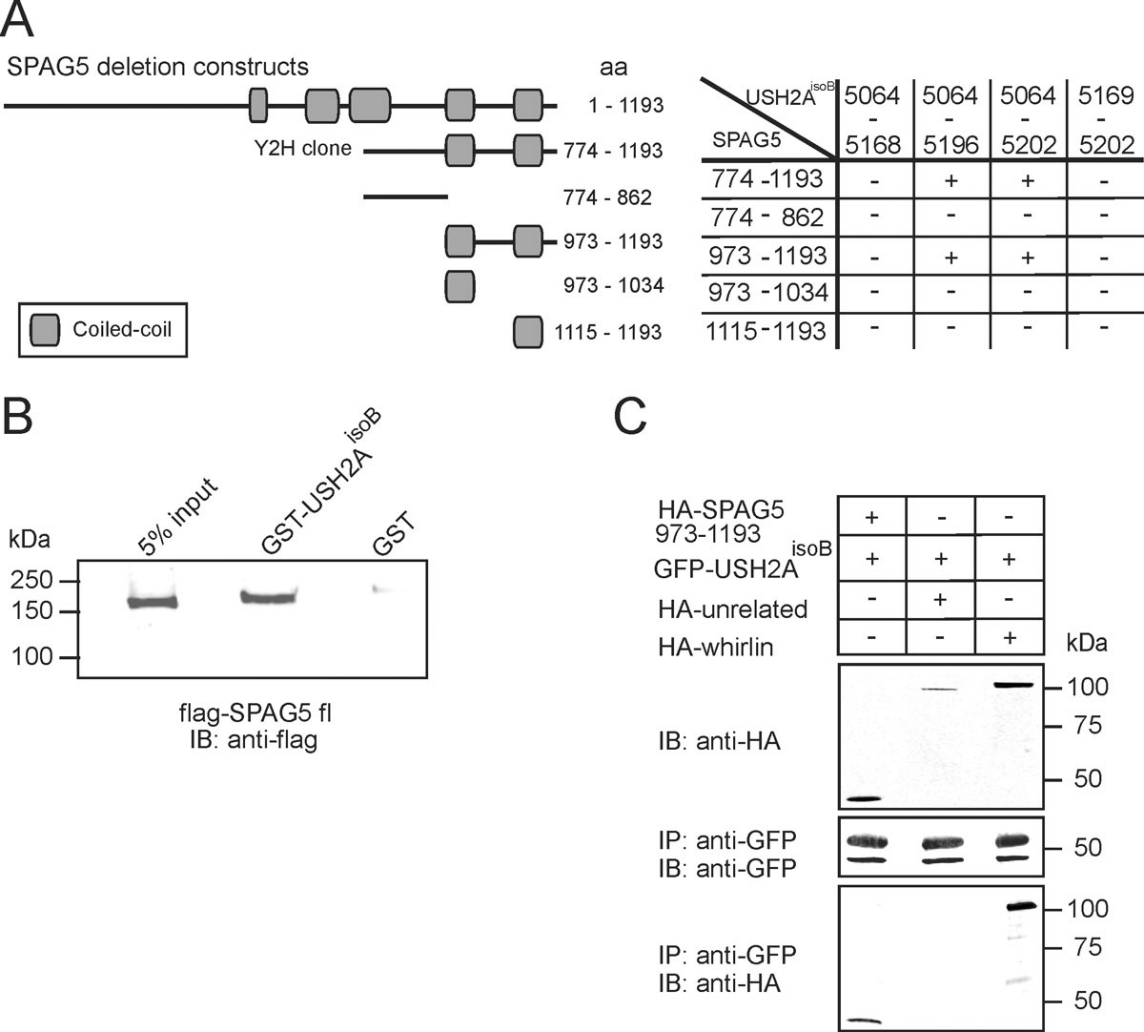
**Interactions between sperm-associated antigen (SPAG)5 and Usher syndrome 2A isoform B (USH2A^isoB^)**. (**A**) Schematic protein structures of SPAG5 and the peptides encoded by deletion constructs. Numbers represent amino acids (aa; NP_006452). Protein fragments encoded by deletion constructs of *SPAG5 *and the intracellular domain (ICD) of USH2A^isoB ^were used in a yeast two-hybrid assay, which identified a specific interaction between USH2A^isoB ^(aa 5064 to 5196) and the C-terminal two coiled-coil domains (aa 973to 1193) of SPAG5. (**B**) Glutathione S-transferase (GST) pull-down assays showing that Flag-tagged SPAG5 is efficiently pulled down by GST-USH2A^isoB^ICD, but not by GST alone, as detected by an anti-Flag antibody. The first lane shows 5% of the input of COS-1 cell lysate. (**C**) Hemagluttinin (HA)-SPAG5 aa 973-1193 from COS-1 lysates co-immunoprecipitated with GFP-fused USH2A^isoB^ICD, but not with an unrelated HA-tagged protein (EPS8). As a positive control, HA-whirlin co-immunoprecipitated with green fluorescent protein (GFP)-fused USH2A^isoB^ICD. The upper protein band in the middle blot represents the heavy chain of the anti-GFP antibody.

A yeast two-hybrid (Y2H) screen of an oligo-d(T) primed human retinal cDNA library was performed to identify interaction partners of USH2A^isoB^, by using its intracellular domain (ICD; USH2A^isoB^ICD) as a bait. From a group of clones that activated all four reporter genes, two identical clones, encoding SPAG5 amino acids (aa) 774 to 1193, were identified (Figure 1A). The interaction between USH2A^isoB ^and SPAG5 was confirmed by a glutathione *S*-transferase (GST) pull-down assay, in which full-length Flag-tagged SPAG5 was efficiently pulled down from COS-1 cell lysates by GST-fused USH2A^isoB^ICD but not by GST alone (Figure 1B). To determine the interacting epitopes of the proteins, we performed an Y2H analysis using constructs encoding fragments of USH2A^isoB^ICD (aa 5064 to 5202) and the SPAG5 Y2H clone. The USH2A^isoB ^peptide containing aa 5064 to 5196 was determined to interact with the SPAG5 fragment containing aa 973 to 1193 (Figure 1A). To further validate the interaction, we performed a co-immunoprecipitation assay from COS-1 cells. This assay showed that hemagglutinin (HA)-tagged SPAG5 aa 973 to 1193 specifically co-immunoprecipitated with the GFP-tagged USH2A^isoB^ICD peptide (Figure 1C).

**Figure 2:**
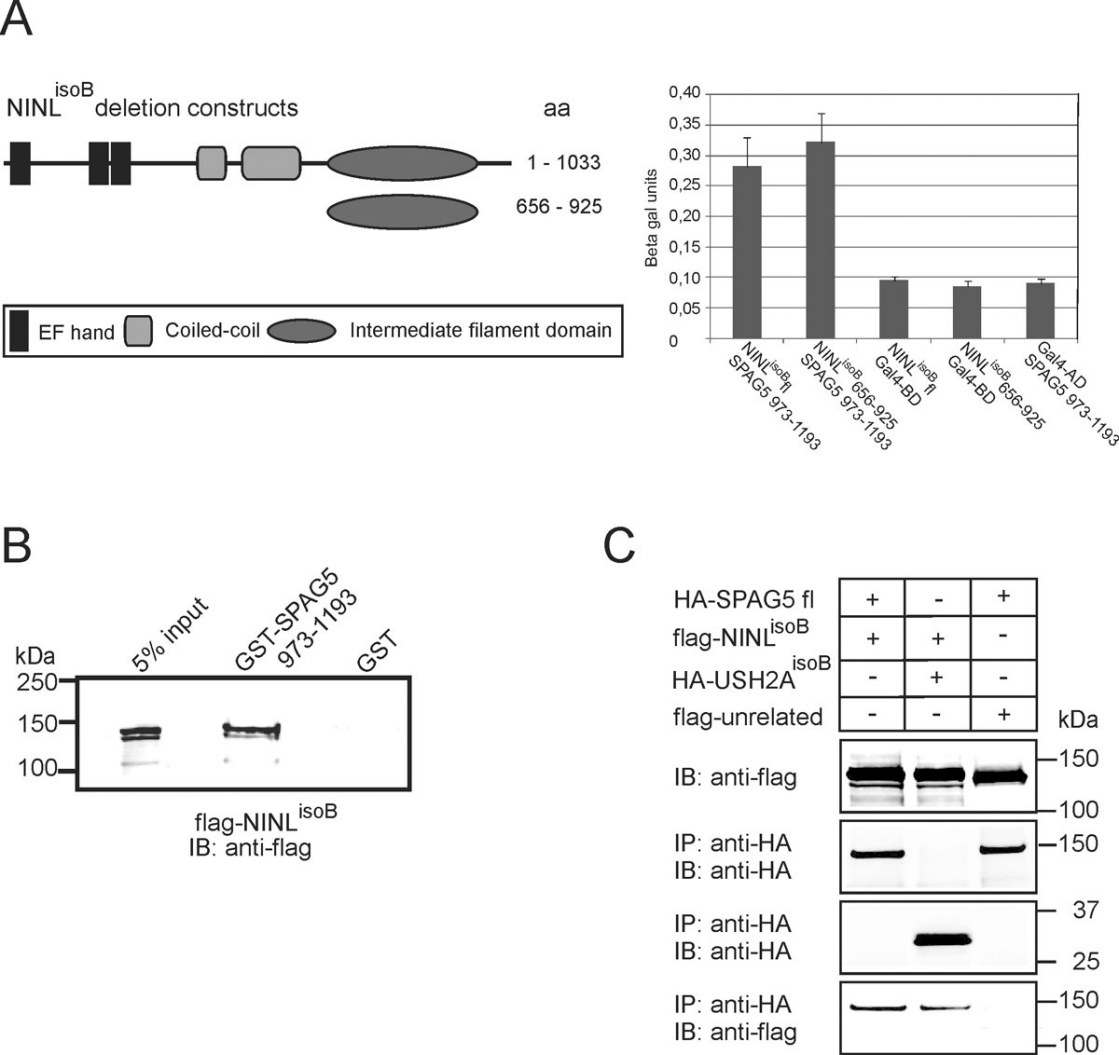
**Validation of the interaction between sperm-associated antigen (SPAG)5 and ninein-like protein isoform B (NINL^isoB^) interaction**. (**A**) The schematic protein structure of NINL^isoB ^and the predicted intermediate filament domain (IF; aa 656-925), encoded by a deletion construct. A liquid β-galactosidase assay identified a specific interaction between SPAG5 (aa 973-1193) and the intermediate filament (IF) domain of NINL^isoB^. (**B**) Glutathione S-transferase (GST) pull-down assay showing that Flag-tagged NINL^isoB ^was efficiently pulled down by GST-SPAG5 (aa 973-1193), but not by GST alone, as detected by an anti-Flag antibody. The first lane shows 5% of the protein input. (**C**) Flag-NINL^isoB ^co-immunoprecipitated with full-length HA-SPAG5 and the positive control HA-USH2A^isoB ^ICD, but not with the HA-tagged unrelated protein MDA5.

In the described Y2H screen with the USH2A^isoB^ICD peptide as bait, NINL^isoB ^was also identified as an interaction partner [13]. Interestingly, Cheng and co-workers previously found that SPAG5 also interacts with ninein [17], which is a paralog of NINL. We therefore assessed the interaction between SPAG5 and NINL also. In a liquid β-galactosidase assay, both full-length NINL^isoB ^and the predicted NINL^isoB ^intermediate filament (IF) domain (aa 656 to 925) were found to interact with SPAG5 aa 973 to 1193 (Figure 2A). The interaction strength between SPAG5 aa 973 to 1193 and full-length NINL^isoA ^was significantly weaker (data not shown). The interaction between SPAG5 and NINL^isoB ^was confirmed by a GST pull-down assay and by co-immunoprecipitation. Flag-tagged full-length NINL^isoB ^was efficiently pulled down from COS-1 lysates by GST-fused SPAG5 aa: 973 to 1193 (Figure 2B), and it also co-immunoprecipitated with HA-tagged full-length SPAG5 (Figure 2C). From these experiments, we concluded that SPAG5 interacts with both USH2A^isoB ^and NINL^isoB^.

### *SPAG5 *expression in human adult tissues and during murine development

**Figure 3:**
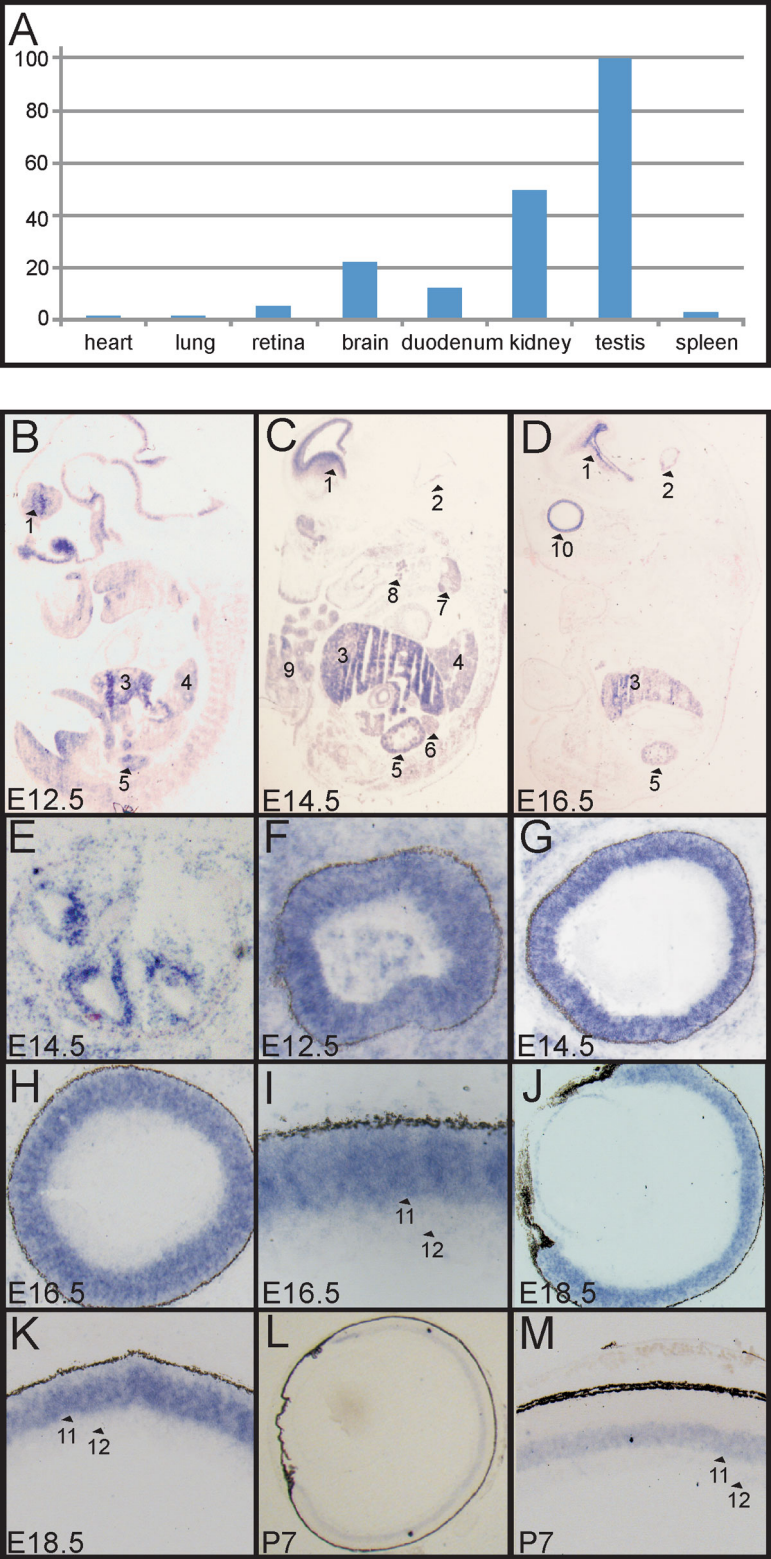
**Expression of the gene s*perm-associated antigen *(*SPAG*)*5 *in human adult tissues and *Spag5 *expression during murine development**. (**A**) Using quantitative PCR, *SPAG5 *expression was detected in the human retina, brain, duodenum, kidney and testis. (**B-M**). RNA *in situ *hybridization of *Spag5 *mRNA in mouse embryos at embryonic day (E)12.5-E18.5 and mouse eyes at postnatal day (P)7. **(B**-**D**) *Spag5 *was widely expressed during murine development (E12.5 to E16.5), with the most intense signals in the following structures (numbers and arrowheads) ventricular zone of forebrain (1), midbrain (2), liver (3), lung (4), kidney (5), adrenal gland (6), thymus gland (7), submandibular salivary gland (8), limbs (9) and eye (10). (**E**) Expression was also seen in the cochlea, primordial vibrissal follicles, upper and lower jaw, intestine, heart, olfactory epithelium and testis (data not shown). **(D) **From E16.5 onwards, expression was mainly detected in the eye, brain, kidney, lung, thymus, primordial vibrissal follicles and in the liver (data not shown). (**F**-**K**) A strong signal for *Spag5 *was seen in the eye during embryonic development E12.5-E18.5 in the neuroblastic layer of the retina. At (**I**) E16.5 and (**K**) E18.5, *Spag5 *expression was present in the developing inner nuclear layer (INL; 11) and, (**L**-**M**) although much weaker, in the ganglion cell layer (GCL; 12), and this was maintained at postnatal day 7.

SPAG5 is known to play a role in cell proliferation, and therefore an important function during development could be expected. To examine this, we determined the expression pattern of *SPAG5 *in human adult tissues by quantitative (q)PCR, using primers in exons 12 and 13. Of the examined human adult tissues, expression of *SPAG5 *was mainly detected in the retina, brain, duodenum, kidney and testis (Figure 3A).

We used RNA *in situ *hybridization (ISH) to determine the expression of *Spag5 *during development. ISH was performed using mouse embryos at embryonic day (E)12.5 to 18.5, and mouse eyes at postnatal day (P)7. *Spag5 *was found to be widely transcribed during murine embryonic development. At E12.5 and E14.5, transcripts were detected in the eye, cochlea, forebrain, midbrain, kidney, liver, lung, adrenal gland, thymus, submandibular (salivary) glands, primordial vibrassal follicles, limbs, upper and lower jaws, intestine, heart, olfactory epithelium, and testes (Figure 3B-C, 3E-G; other data not shown). From E16.5 onwards, *Spag5 *transcripts were mainly present in the eye, brain, kidney, lung, thymus, liver, and primordial vibrassal follicles (Figure 3D, H-K; other data not shown). From E12.5 to E18.5, *Spag5 *transcripts were seen in the neuroblastic layer of the retina (Figure 3F-K), and at E16.5 (Figure 3I) and E18.5 (Figure 3K) these transcripts were detected more specifically at the (developing) inner nuclear layer (INL) and in low amounts in the ganglion cell layer (GCL). This pattern was maintained at P7 (Figure 3L-M). Hybridizations with a sense cRNA probe did not detect any signal, confirming the specificity of the assay (data not shown).

### Spag5 localization in the retina

**Figure 4:**
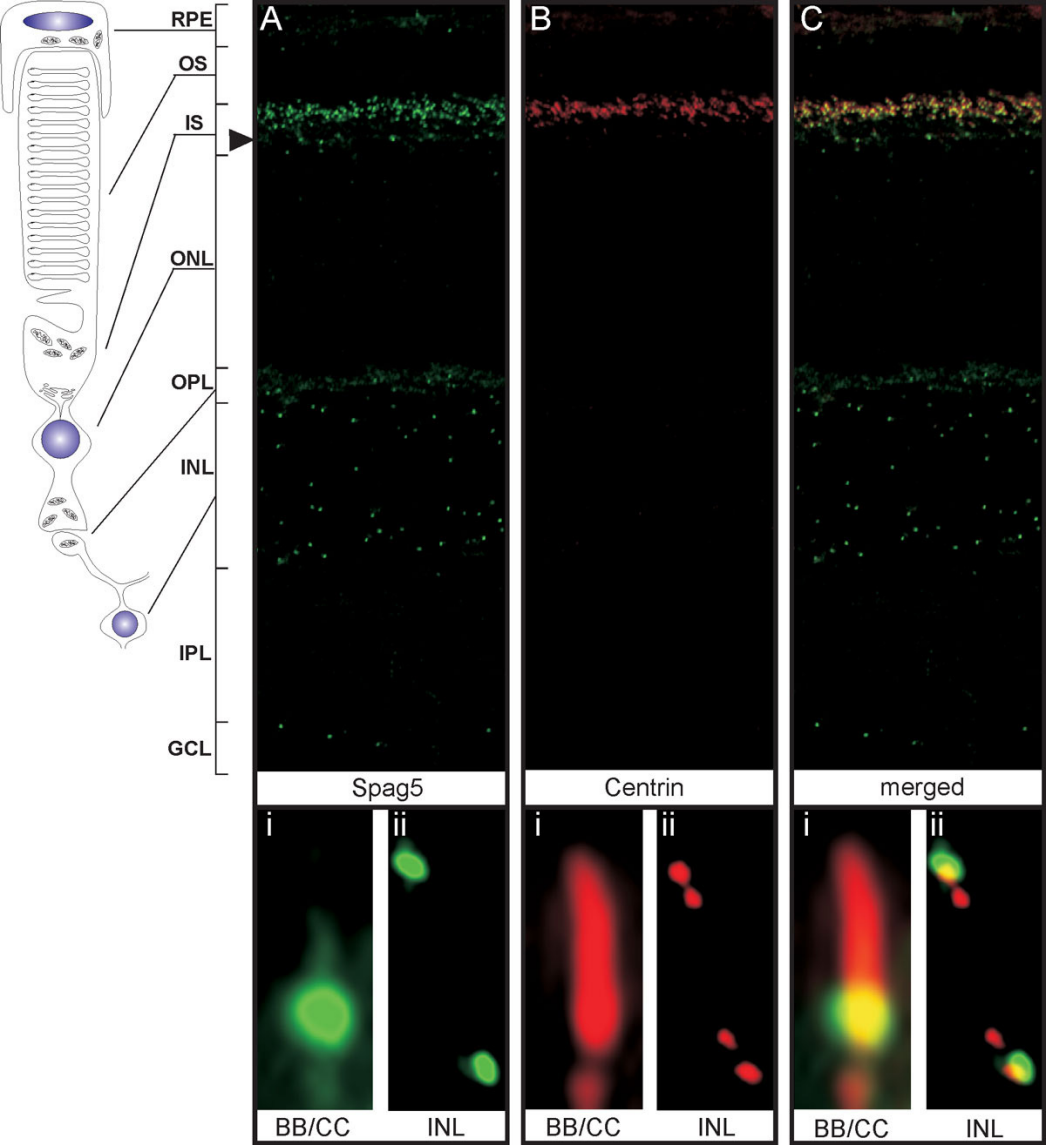
**Subcellular localization of sperm-associated antigen (Spag)5 in retina cryosections of adult (postnatal day (P)20) rats**. (**A**-**C**) Co-immunostaining using**(A) **anti-SPAG5 (green) and **(B) **anti-pan-centrin (red, a marker for the connecting cilium, basal body and centrioles) antibodies. **(C) **Colocalization (**C**; yellow) was detected at the **(Ci) **basal body (BB) and **(Cii) **in one of the two centrioles of the inner nuclear layer (INL). **(A,C) **Centriole staining is also visible in the ganglion cell layer (GCL). In addition, weaker Spag5 labeling was detected at the outer limiting membrane (OLM; filled arrowhead), the outer plexiform layer (OPL), at the distal part of the connecting cilium (CC) and at the accessory centriole. (**A-Ci**-**ii**).High-magnification fluorescence microscopy of the BB/CC region and the INL.

Because SPAG5 was identified from a retinal cDNA library, and *Spag5 *mRNA was expressed in the developing and the adult retina, we set out to determine its retinal subcellular localization. For this purpose, rat cryosections (P20) were co-stained with affinity-purified anti-SPAG5 antibodies (green; Figure 4A-Aii) and monoclonal anti-*pan-*centrin antibodies (20H5, red; Figure 4B-Bii) as markers for the basal body, connecting cilium, accessory centriole of photoreceptor cells, and centrioles in both the INL and the GCL [18]. Spag5 was predominantly detected at the basal body of photoreceptor cells, and at one of the two centrosomal centrioles in both the INL and GCL, as shown by partial colocalization with centrins that mark these structures (yellow; Figures 4C-Cii; other data not shown). In addition, Spag5 immunostaining was weakly detected at the distal part of the connecting cilium (CC; Figure 4A-Ai open arrowhead), in the accessory centriole, at the outer limiting membrane (OLM; Figure 4A-Ai, filled arrowhead) and at the outer plexiform layer (OPL) (Figure 4A-Ai).

**Figure 5:**
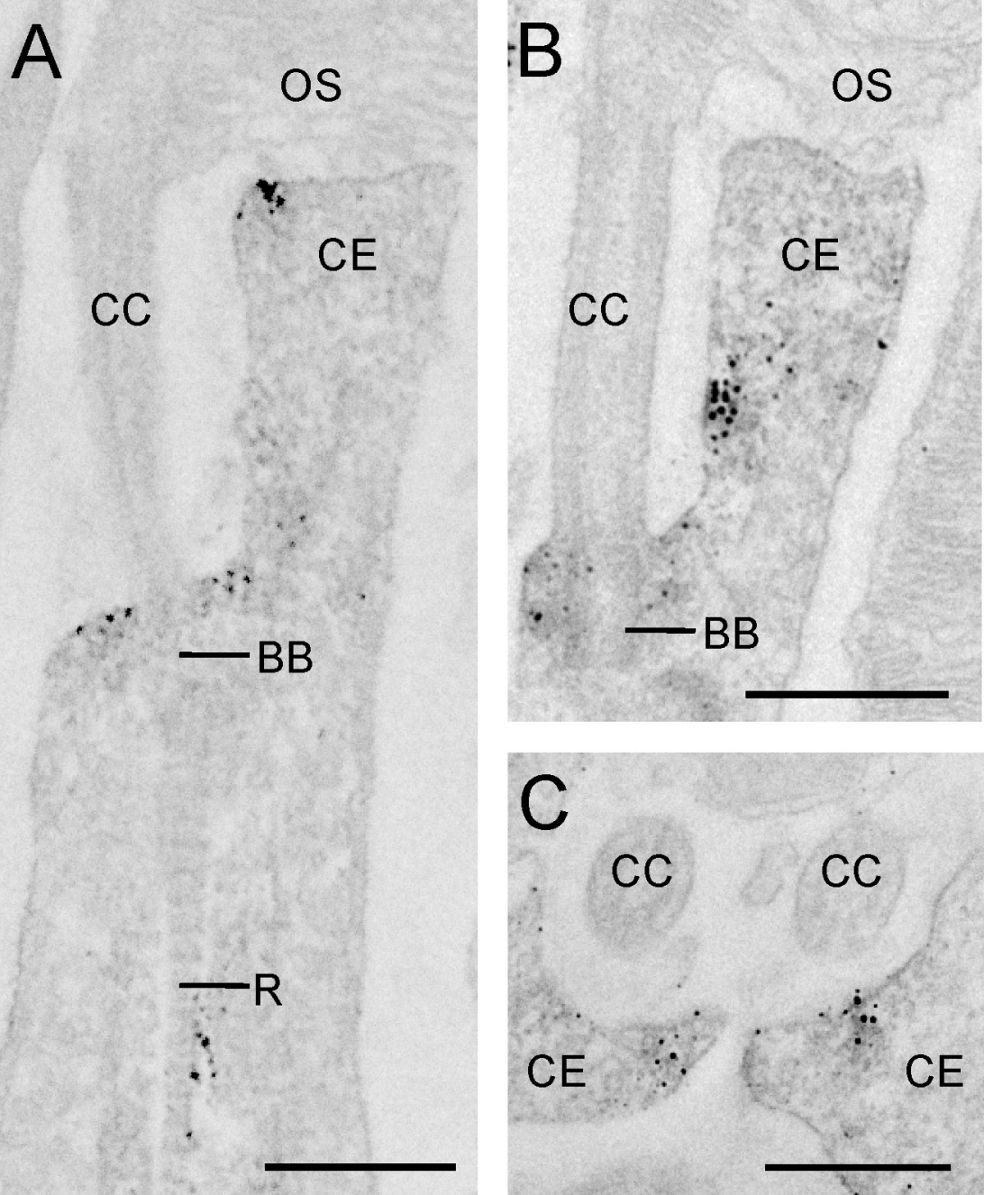
**Localization of sperm-associated antigen (Spag)5 by immunoelectron microscopy**. Electron micrographs of pre-embedded anti-SPAG5 labeling in (**A**,**B**) longitudinal and (**C**) cross-sections of mouse rod photoreceptor cells. Spag5 was detected **(A**-**B) **around the basal body (BB), **(A**-**C) **in the collar-like extension (CE) of the apical inner segment; and **(A) **in some photoreceptors at the region of the rootlet (R). CC = connecting cilium; OS = outer segment; IS = inner segment. Scale bars: 0.5 μm.

To determine the Spag5 distribution in the photoreceptor cells in more detail, we used immunoelectron microscopy. Spag5 was detected around the basal body (BB; Figure 5A-B) and at the collar-like extension (CE) of the apical inner segment (Figure 5A-C). In some photoreceptors, labeling was detected in the region of the rootlet (R; Figure 5A). In previous studies, immunoelectron microscopy showed subcellular localization of Ush2a^isoB ^and Ninl^isoB ^at these structures as well, although both proteins were detected exactly at the basal body [10,13], whereas Spag5 was mainly located around it.

### Spag5 colocalization with Ush2a^isoB ^and Ninl^isoB ^in photoreceptor cells

**Figure 6:**
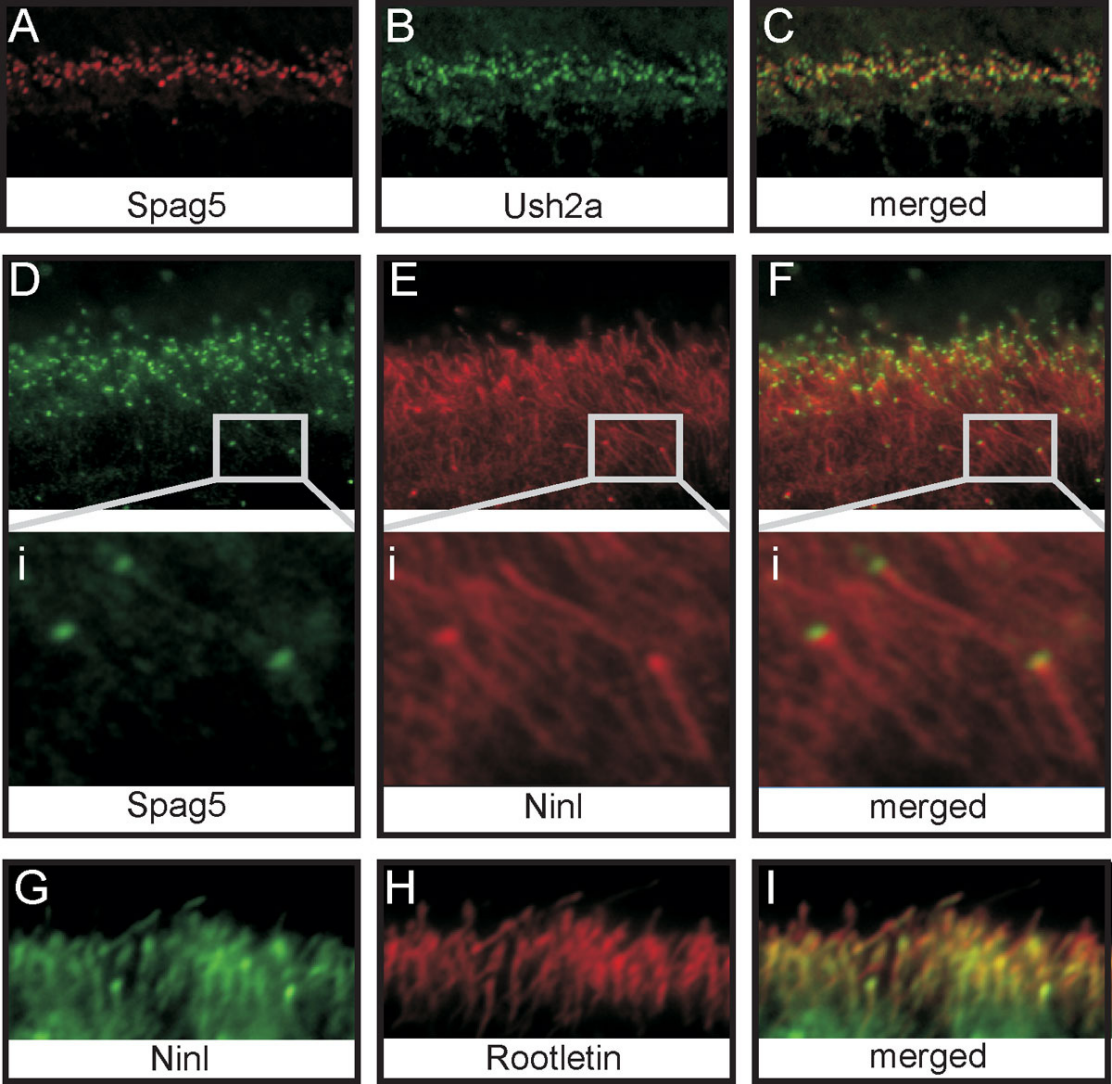
**Colocalization of sperm-associated antigen (Spag)5 and isoform B of Ush2a (Ush2a^isoB^) and ninein-like (NINL^isoB^) proteins in the retina of young adult (P20) rat**. Co-immunostaining of Spag5 and Ush2a^isoB ^in retinal cryosections using **(A) **anti-SPAG5 antibodies (red) and **(B) **anti-USH2A^isoB ^antibodies (green) show **(C) **partial colocalization (yellow) at the basal body. Co-immunostaining using **(D) **anti-SPAG5 antibodies (green) and **(E) **anti-NINL^isoB ^antibodies (red). **(F) **A partial colocalization (yellow) was detected at the basal body. In addition, both antibodies labeled **(Di,Ei) **the ciliary rootlets, which was confirmed by **(I) **co-staining (yellow) using **(G) **anti-NINL^isoB ^(green) and **(H) **anti-rootletin (red) antibodies.

To investigate whether Spag5 and Ush2a^isoB ^colocalize in the retina, we performed immunostaining on retinal cryosections using affinity-purified antibodies against SPAG5 (red; Figure 6A) and USH2A^isoB ^(green; Figure 6B), which revealed partial colocalization of these proteins at the basal body (yellow; Figure 6C). The same analysis was performed for SPAG5 and NINL^isoB^. Using affinity-purified antibodies against SPAG5 (green; Figure 6D) and NINL^isoB ^(red; Figure 6E), we detected partial colocalization at the basal body/centriole in the region of the connecting cilium and in the INL (yellow; Figure 6F and data not shown). Although staining was weak for anti-SPAG5, both antibodies also stained the ciliary rootlets (Figure 6Di-Ei). The rootlet localization of SPAG5 was confirmed indirectly by co-immunostaining (yellow; Figure 6I) using antibodies against NINL^isoB ^(green; Figure 6G) and rootletin (red; Figure 6H).

## Discussion

In this study, we identified SPAG5 as a novel member of the Usher protein network via its interaction with both USH2A^isoB ^and NINL^isoB^. SPAG5 is a non-motor microtubule-associated protein that is essential for progression through mitosis [15,16,19,20]. We found that *Spag5 *is widely expressed during mouse development, which is consistent with its function in cell proliferation. However, we also detected Spag5 in photoreceptor cells, indicating that Spag5 also plays a postmitotic role in differentiated ciliated cells.

During mitosis, SPAG5 localizes to centrosomes and to spindle and kinetochore microtubules, and is required for correct mitotic spindle formation, sister chromatid cohesion and centrosome integrity [15,16,19-21]. By contrast, the interaction partner of SPAG5, NINL^isoB^, mainly fulfills a role during interphase, in which it is involved in microtubule organization and nucleation [22-24]. However, during cytokinesis at the end-stage of mitosis, SPAG5 and NINL are detected at the midzone microtubules [15,21] and the midbody [13,25], respectively. Depletion of SPAG5 results in cellular growth arrest with disorganized multipolar spindles, which ultimately leads to (p53-dependent) apoptosis [16,20,26]. NINL is required for proper centrosome maturation and spindle assembly, and NINL abnormalities may contribute to genomic instability and tumorigenesis [22,27,28]. Hence, both SPAG5 and NINL are essential for proper cell proliferation.

After exit from the cell cycle, a primary cilium develops, which essentially is a microtubule-based antenna-like structure that protrudes from the (apical) surface of many cell types. Cilia are involved in a wide variety of sensory functions, such as chemical, mechanical and photo sensation, Hedgehog signaling, and control of cell growth [29,30]. Cilia assembly and disassembly are closely coupled to the cell cycle [31]. Therefore, it is not surprising that similar protein complexes play a role in both cell-cycle regulation and cilia assembly and function. Indeed, Smith and co-workers recently found that the proteomes of the midbody and of the cilia/basal body significantly overlap [32]. Furthermore, they reported the presence of a number of central spindle/midbody proteins, which control cytokinesis during mitosis, at the basal body/rootlet of ciliated cells, where they were suggested to control cilia morphogenesis and function [32].

The outer segment of the postmitotic photoreceptor cell is a highly specialized sensory cilium, with the connecting cilium as the ciliary transition zone [33,34]. Both the proteins involved in the formation and maintenance of the outer segments and the proteins involved in the visual cycle are synthesized in the inner segment, and actively transported to the outer segment via involvement of the IFT machinery [14,35]. A significant part of protein transport towards the basal body is via *trans*-Golgi derived vesicles that migrate along microtubules. These vesicles are thought to dock in the region of the basal body and at the membrane of the apical inner segment that surrounds the connecting cilium as a collar [10,14,36,37]. At the central spindle and midbody, a protein network, including proteins that are also present at the basal body, organizes the microtubule bundles and vesicle recruitment for cell abscission [32]. Based on this and on the (co)localization of Spag5 and Ninl^isoB ^at the basal body, the rootlet and the collar-like apical inner segment of photoreceptor cells (Figure 5, Figure 6) [13], we propose that both proteins function in the development and/or function of the photoreceptor cilium by direct involvement in vesicle transport towards the basal body and possibly the rootlet, or by an indirect role in microtubule organization. More specifically, Ninl^isoB ^might function as an adapter protein in this retrograde transport through its association with the dynactin p50-dynamitin and p150^Glued ^subunits of the dynein-dynactin motor complex [13,23]. This complex also transports kinetochore-components from kinetochores to spindle poles during mitosis [38]. As Spag5 is present at kinetochores and spindle poles during mitosis [15,16], it may serve as cargo of this complex. Therefore, Spag5 may be transported towards the basal body by the dynein-dynactin motor complex via its interaction with Ninl^isoB^, analogous to the targeting of Spag5 to the centrosome by the Ninl paralog, ninein, during the cell cycle [17]. At the basal body, Spag5 may have a structural and organizational role by bundling microtubules or crosslinking them to other components, similar to its role during mitosis [15,16]. Because both SPAG5 and NINL^isoB ^associate with the cytoplasmic region of USH2A^isoB^, these proteins may well be involved in microtubule-based transport of USH2A^isoB^-containing vesicles towards the membrane of the apical inner segment of photoreceptor cells. Indeed, Zallocchi and co-workers provided evidence for the existence of USH protein complexes in vesicles of tracheal epithelium, and their results implied that the ectodomain of Ush2a^isoB ^faces the lumen of vesicles, with its cytoplasmic domain towards the cytosol [39].

The association of SPAG5 with both USH2A^isoB ^and NINL^isoB ^and its localization in photoreceptor cells pinpoints *SPAG5 *as a functional candidate gene for retinal ciliopathies. This hypothesis is supported by the observed expression of *Spag5 *in tissues such as retina, kidney, brain and testis, which are often affected in ciliopathies.

Two *Spag5 *animal models have been reported. *Spag5*-null mice were found to be viable and fertile, and did not display a clear phenotype [40]. However, defects may have been missed, as this model is probably phenotyped with a focus on spermatogenesis and fertility anomalies. The rat model is characterized by male sterility, reduced female fertility, hypogonadism, progressive renal insufficiency and body growth retardation, as a consequence of hypogenesis that might result from defective cell proliferation [41,42]. The renal phenotype is similar to oligomeganephronia [43,44], the most common form of human congenital renal hypoplasia, which in a few cases is found to be associated with RP, hearing impairment and stunted growth [45]. *SPAG5 *is a candidate gene for association with the clinical features in the patient described by Janin-Mercier and co-workers [45]. No retinal dysfunction has been reported in the *Spag5 *animal models, thus a detailed retinal examination is needed to exclude defects in visual function; unfortunately, the models were no longer available for this analysis.

## Conclusions

In conclusion, our studies show that SPAG5 associates with both USH2A^isoB ^and NINL^isoB^. Therefore, it seems likely that besides its known function in cell proliferation, SPAG5 has a role in differentiated cells such as photoreceptor cells. Our data suggest that SPAG5 and NINL^isoB ^function directly or indirectly in the microtubule-based transport and docking of (USH2A^isoB^-containing) vesicles to the apical inner segment and basal body of photoreceptor cells, which is essential for their long-term maintenance and/or function. In continuation with its expression pattern, this indicates that *SPAG5 *is a very likely candidate gene for (retinal) ciliopathies.

## Methods

### Animals

The Wistar rats and C57BL6 JOlaHsD mice (Harlan, Horst, The Netherlands) used in this study were housed in standard cages and received water and food *ad libitum*. All experiments were conducted according to international and institutional guidelines.

### Plasmids and antibodies

The human image clone IRAUp969C082D (ImaGenes.GmbH, Berlin, Germany) was used as a template to amplify full-length SPAG5. The amino acids were numbered according to the entries in GenBank (NP_006452 (SPAG5), NP_996816 (USH2A), EU718622) (NINL^isoB^; https://www.ncbi.nlm.nih.gov/protein; provided in the public domain by the National Center for Biotechnology Information, Bethesda, MD) All constructs were generated using commercial cloning technology (Gateway; Invitrogen, Carlsbad, CA, USA), according to the manufacturer's instructions.

**Figure 7:**
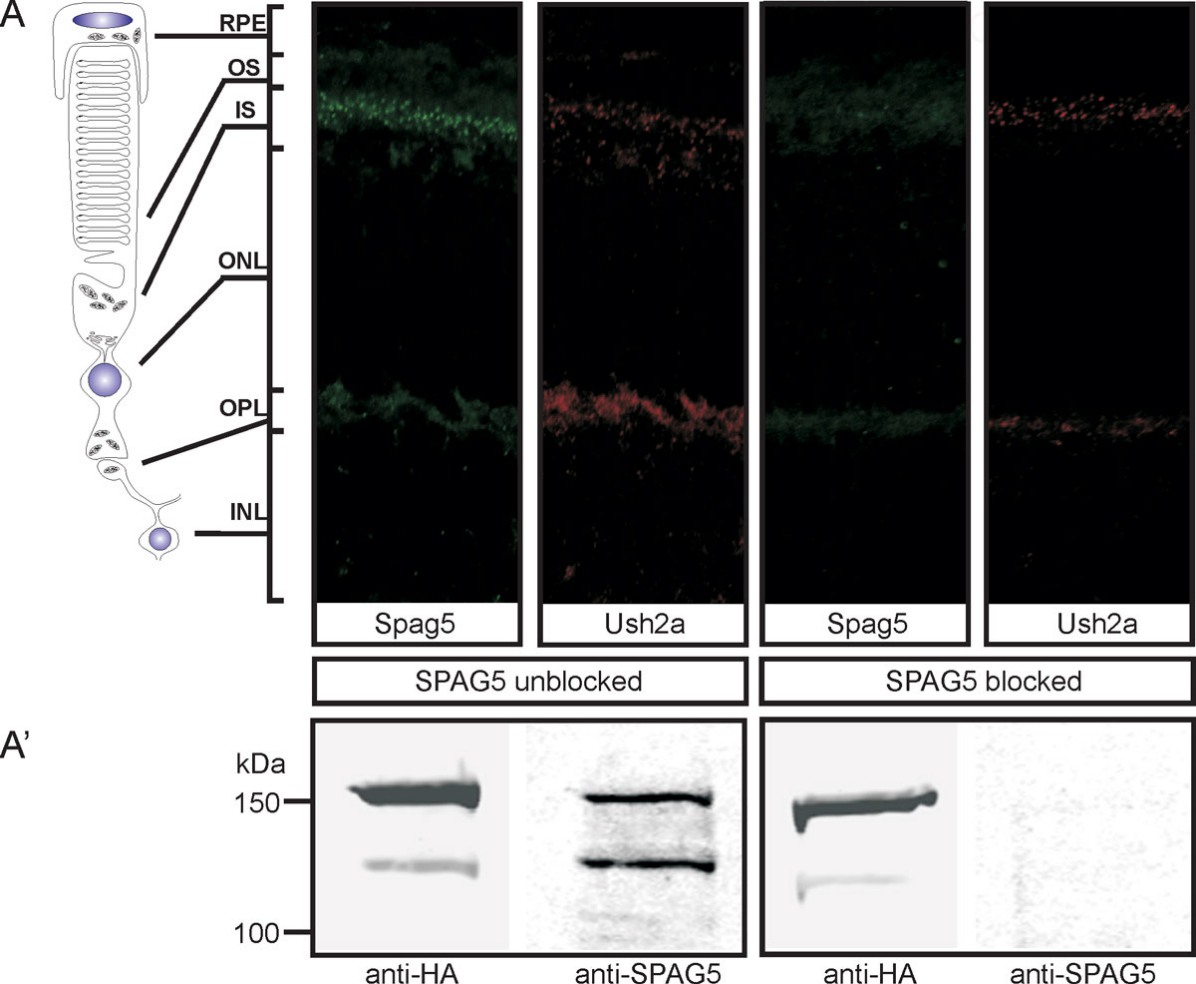
**Specificity of anti-sperm-associated antigen (SPAG)5 antibodies**. (**A**) Co-immunostaining of SPAG5 (green) and Usher syndrome 2A (USH2A; red) in retinal cryosections of adult (P20) rat using anti-USH2A and anti-SPAG5 antibodies. Both antibodies were blocked together with the SPAG5 antigen and after pre-absorption no specific SPAG5 signals were detected in contrast to the normal USH2A staining. (**A'**) Western blot analysis of HA-tagged SPAG5 using (pre-absorbed) anti-hemagglutinin (HA) and anti-SPAG5 antibodies. After pre-absorption of the antibodies with the SPAG5 antigen, HA-SPAG5 could only be detected by anti-HA antibodies and not by the blocked anti-SPAG5 antibodies, indicating the specificity of the antibodies.

Antibodies against the C-terminal region of SPAG5 were used, which were raised in guinea pigs against a GST-fusion protein encoding a peptide consisting of aa 1063 to 1187. The cDNA encoding this peptide was amplified by using the forward and reverse primers 5'-GGCGAGCTCATAAGCCTTAG-3' and 5'-TCCCTGTAGTTCTTTGC-3', respectively. The affinity-purified SPAG5 antibodies did not stain tissue immunohistochemically and they did not detect SPAG5 on western blot after pre-adsorption of the primary antibodies with the antigen (Figure 7). Antibodies against rootletin (Novus Biologicals, Cambridge, UK) were used at 1:1000 dilution. The monoclonal antibodies directed against centrins, and the polyclonal antibodies directed against NINL and the cytoplasmic region of USH2A^isoB ^have been described previously [12,13,46]; all were used at 1:250 dilution. Anti-HA and anti-Flag (Sigma-Aldrich, Munich, Germany) were used at 1:1000 dilution. The secondary antibodies were goat anti-guinea pig Alexa Fluor 488 and Alexa Fluor 568, goat anti-rabbit Alexa Fluor 568, goat-anti-human Alexa Fluor 568, IRDye800 goat anti-guinea pig IgG, IRDye800 goat-anti-mouse, and IRDye800 goat anti-rabbit (all used at 1:500 dilution, and all from Molecular Probes-Invitrogen Carlsbad, CA, USA).

### Yeast two-hybrid analysis

A GAL4-based yeast two-hybrid system (HybriZAP, Stratagene, La Jolla, CA, USA) to identify proteins that interact with the cytoplasmic region of USH2A^isoB ^(aa 5064 to 5202; NP_996816) was used as previously described [13,47].

### GST pull-down

The GST-fusion proteins were produced by transforming *Escherichia coli *BL21-DE3 with plasmids pDEST15-USH2A_ICD (aa 5064 to 5202) or pDEST15-SPAG5 (aa 973 to 1193) respectively, as previously described [12]. Flag-tagged SPAG5 or Flag-tagged NINL^isoB ^were produced by transfecting COS-1 cells with plasmids encoding p3xFlag-SPAG5 or NINL^isoB^, respectively, using a transfection reagent (Effectene; Qiagen, Hildene, Germany) according the manufacturer's instructions. The GST pull-down assay was performed as described previously [12].

### Immunohistochemistry in rat retina

Unfixed eyes removed from 20-day-old (P20) Wistar rats were isolated and frozen in melting isopentane. Cryosections 7 μm thick were treated and immunolabeled as described previously [12]. Immunofluorescence was visualized under a fluorescent microscope (Axio Imager Z1; Zeiss, Basle, Switzerland) equipped with a camera (AxioCam MRm; Zeiss). Images were processed using Axiovision Rel (version 4.6) and Adobe Photoshop (Adobe Systems, San Jose, CA, USA).

### Expression by real-time quantitative PCR

Total RNA was used from several human adult tissues (Agilent Technologies, Santa Clara, CA, USA) and cDNA was generated using reverse transcriptase according the manufacturer's instructions (iScript; Bio-rad laboratories, Hercules, CA, USA). cDNA primers were designed using Primer3 and validated for SPAG5 and the reference gene glucuronidase-β (GUSB) (primers: SPAG5 exon 12 to 13 forward 5'-ACTCTGCCAGCTTACCCAG-3' and SPAG5 reverse 5'-CAGTTCTGCCTGCATGTG-3', GUSB forward 5'-AGAGTGGTGCTGAGGATTGG-3' and GUSB reverse 5'-CCCTCATGCTCTAGCGTGTC-3). Quantitative (q)PCR was performed using adult human cDNA as a template and the SYBR Green PCR master mix (Applied Biosystems, Life Technologies, Foster City, CA, USA). PCR program: at 95°C for 10 minutes, 40 cycles at 95°C for 15 seconds and 60°C for 30 seconds, followed by 60 cycles at 95°C for 1 minute, 65°C for 1 minute (7500 Fast Real-Time PCR System; Applied Biosystems). Data were analyzed using the system software SDS (version 1.4.0.25).

### **Mouse RNA probes *in situ *hybridization**

A probe corresponding to nucleotides 2698 to 3757 [GenBank: NM_017407], which recognizes mouse *Spag5 *transcripts was generated from mouse retina cDNA (Marathon; Clontech, USA) with the forward and reverse primers 5'-ATACCTGTGCAGGCTGGAG-3' and 5'-GTTCTCTTCCAAGTCGTGTC-3', respectively. Digoxigenin labeling was performed as previously described [12]. C57Bl6 Jlco mouse embryos were collected at various embryonic stages (E12.5 to E18.5), and mouse eyes at P7. RNA *in situ *hybridization was performed as previously described [12].

### Pre-embedding immunoelectron microscopy

Vibratome-cut sections of mouse retina were incubated with a an antibody (1 100 dilution) against SPAG5 and visualized by a secondary antibody (Vectastain ABC Kit, Vector Laboratories, Peterborough, UK). After fixation with 0.5% OsO_4_, the specimens were embedded in araldite, and ultrathin sections were viewed with a transmission electron microscope (Tecnai 12; FEI, Hillsboro, OR), as described previously [10].

## List of abbreviations

aa: Amino acid; BB: basal body; CC: connecting cilium; GCL: ganglion cell layer; GST: glutathione *S*-transferase; INL: inner nuclear layer; ICD: intracellular domain; IFT: intraflagellar transport; NINL^isoB^: ninein-like protein isoform B; RP: retinitis pigmentosa; USH2: Usher syndrome type II;: yeast two-hybrid.

## Competing interests

The authors declare that they have no competing interests

## Authors' contributions

FFJK carried out a major part of the experimental work, and was the primary author of the manuscript. EvW was closely involved during the whole process including some of the experiments, and contributed to writing the manuscript. LH carried out the real-time qPCR assays and was involved in a number of the other experiments, KB carried out the immunoelectron microscopy experiment, TAP was involved in the immunohistochemistry assays, MGA was involved in the protein-protein interaction experiments, and BvdZ performed a major part of the work for the *in situ *hybridization data. UW supervised the immunoelectron microscopy work and co-edited the manuscript. JEEK was co-supervisor of the PhD project, co-applicant of the major grant that supported this research, and critically read the manuscript. RR was co-supervisor of the study, co-applicant of grants that supported this research, and critically read the manuscript. HK was the primary supervisor of the project, co-applicant of the grants that supported this research, and contributed to the writing of this the manuscript. All authors read and approved the final manuscript.
